# Horseradish peroxidase encapsulation in alginate microspheres in the presence of imidazolium ionic liquids

**DOI:** 10.1186/1753-6561-8-S4-P191

**Published:** 2014-10-01

**Authors:** Flávia Michelle Santos, Raiane Maiara dos Santos, Micael Melo, Heiddy Alvarez, Álvaro Lima, Cleide Mara Soares, Alini Fricks

**Affiliations:** 1 Universidade Tiradentes, Instituto de Tecnologia e Pesquisa, Aracaju, SE, Brasil; 2 Universidade Tiradentes, Aracaju, SE, Brasil; 3 Universidade Estadual de Feira de Santana, Departamento de Ciências Exatas, Feira de Santana, BA, Brasil

## 

Biocatalysis with free enzymes may not be economically viable due to the complexity of their recovery in the reaction medium. With the objective of enabling the total activity recovery yield of biocatalyst and to improve the operational characteristics, have been applied enzyme immobilization techniques. The main interest in the enzyme immobilization is to obtain a catalyst whose activity and stability is not affected during the process when compared to the free enzyme. The objective of this work was to immobilize horseradish peroxidase (HRP) by encapsulation method in alginate microspheres in the presence of imidazolium ionic liquid (ILs). The enzyme encapsulation was carried with the enzyme incorporation in an aqueous solution of sodium alginate to 0.05 % (w / v). The influence of the enzyme loading was studied in the range of 0.0406-0.65 mg HRP / g alginate. Alginate microspheres were obtained by dripping in calcium chloride solution (1M) according to literature [1]. The HRP immobilization in the presence of ILs was conducted using the enzyme loading more appropriate, incorporating in the immobilization medium 1% (w/v) of IL. The ILs studied as additives were: [C_4_mim]TF_2_N; [C_4_mim]BF_4_; [C_4_mim]HSO_4_; [C_4_mim]Ac e [C_4_mim]PF_6_. The activity of free and immobilized enzyme was verified by colorimetric method based on the change of absorbance at 470 nm due to the formation of product in guaiacol oxidation for three minutes. The results obtained for biocatalysts immobilized by encapsulation suggest that the use of IL as an additive in the immobilization process causes a significant increase in the total activity recovery yield (RA) HRP (increase from 5.2 to 23.5 %). The results for the HRP immobilization showed that RA was 23.5% and 20.40% for the LIs [C_4_mim] HSO_4 _and [C_4_mim]TF_2_N, respectively. [C4mim]Ac provided RA of 13.3 %, while the biocatalyst with [C4mim] PF_6 _showed RA of 17.3 %. The [C4mim] BF_4 _showed the lowest performance as an additive among the ILs studied. According to Diego et al.(2009) [2] there is not have a rule to predict the behavior of immobilized enzymes in the presence of ILs. The results obtained for biocatalysts immobilized by encapsulation in alginate microspheres show that the use of ionic liquids in this process had a positive effect on the activity of the immobilized biocatalyst. Additional studies in the structural characteristic of the immobilized biocatalyst are being conducted to evaluate the influence of ILs in the supporting structure.
